# Australian Hard 2 (AH2) wheat class with null4A genetic basis to produce high‐quality Japanese ramen noodle

**DOI:** 10.1002/jsfa.70327

**Published:** 2025-11-19

**Authors:** Siem Doo Siah, Yousuke Shinkai, Meiqin Lu, James Edwards, Ken Quail, Hideki Okusu

**Affiliations:** ^1^ Australian Export Grains Innovation Centre North Ryde NSW Australia; ^2^ NIPPN Corporation Food Research Center Atsugi Japan; ^3^ Australian Grains Technologies Narrabri NSW Australia; ^4^ Australian Grains Technologies Roseworthy SA Australia

**Keywords:** null4A, hard wheat, noodle, Japanese ramen, amylose, amylopectin

## Abstract

**BACKGROUND:**

Australia exports wheat to various countries for the production of staple foods, including noodles. The Japanese market considers Australian Prime Hard (APH) wheat to be the benchmark for high‐quality ramen noodle production. We hypothesie that hard wheat with relatively low protein content and null4A gene is particularly well‐suited for high‐quality ramen.

**RESULTS:**

Australian Hard 2 (AH2) Class with the null4A gene is shown to produce ramen with sensory attributes comparable to those of APH, based on tests conducted across three seasons with 32 wheat varieties harvested from Queensland or Northern New South Wales, and South Australia. The null4A population has a lower amylose content and improved pasting properties, resulting in ramen with superior sensory attributes, particularly elasticity, compared to the wild‐type. However, the relationship between amylose content and other physicochemical properties, as well as noodle sensory attributes such as firmness, elasticity, surface smoothness and hot soup stability, is not linear. This complexity is explained by the notable range of physicochemical and sensory properties within the null4A population, which are affected by variations in starch and protein qualities, as well as their interactions. Wheat varieties produced in South Australia are largely made up of null4A genetic background and tend to have a higher ash content but exhibit a relatively lower level of speck contamination on raw noodle sheets.

**CONCLUSION:**

AH2, primarily composed of the null4A gene, has potential to produce ramen that appeals to the Japanese market, providing Japan with an alternative to APH for traditional ramen © 2025 The Author(s). *Journal of the Science of Food and Agriculture* published by John Wiley & Sons Ltd on behalf of Society of Chemical Industry.

## INTRODUCTION

Australia produced an average of 26.6 million metric tonnes (mmt) per annum of wheat over the past 10 years (2013–2024), with 22.5 mmt exported in the 2023–2024 period.^1^ Japan is a significant export market for Australian wheat, importing nearly 1 mmt annually over the last 5 years. According to consumer interviews conducted in 2020 by the Japanese Ministry of Agriculture, Forestry and Fisheries, 17% or close to 1 mmt of the total wheat usage (5.75mmt) in Japan is utilised for ramen noodles.

Wheat noodles are made by first creating a noodle dough or crumbs through mixing flour with water. The dough is then pressed through rollers set at various gaps to achieve the desired thickness, before being cut to the final width and length. Noodles are commonly grouped into white salted noodles (WSN) when table salt is added in the formulation, and yellow alkaline noodles (YAN) when alkaline salt and/or sodium chloride is added in the formulation.^2^ Ramen noodles are one of the commonly consumed noodle types in Japan, requiring a bright colour with freedom from speck contamination, with textural mouthfeel properties described as having a firm and elastic bite, and maintaining a good textural integrity and stability when served in hot soup.

Sensory evaluation is a subjective measurement but is still considered crucial for Japanese flour mills in determining the quality of flour used for noodle products.^3^ Ramen is the primary YAN type of noodle consumed in Japan, and the flour used in ramen production frequently undergoes sensory evaluation as part of the quality assessment process.

In the assessment of ramen noodles, they are typically served in a standard hot broth to replicate the conditions under which consumers would enjoy them. Sensory textural stability refers to the ability of the noodles to maintain their integrity and textural characteristics in hot soup over time. Two key attributes in this evaluation are firmness, defined as the resistance or bite of the noodle, and elasticity, characterised by the bounciness or springiness felt when chewing the strands.

In addition to textural properties, ramen is generally expected to be bright in colour, free from specks and have a smooth surface.^2^ Noodle colour is commonly measured objectively using a tristimulus colour meter,^4^ which measures brightness (L* value), red–green (a* value) and yellow–blue (b* value) coordinates. Another important noodle quality measure is noodle colour stability because noodles, especially fresh ones, are subject to time‐dependent discolouration or darkening driven by enzymatic reactions and protein oxidation, aggravated by the presence of specks, which negatively influence consumer perception.^5^, ^6^

The desirable eating properties of YAN are attributed to wheat, for which the physicochemical properties are largely affected by its major components, which consist of about 60–70% starch and 8–15% protein content.^7^ Wheat with higher protein content and quality tends to produce firmer noodles.^8^, ^9^

Starch consists primarily of long chains of amylose and branched structures of amylopectin.^10^ The composition of wheat starch is influenced by genetic factors and environmental conditions, showing greater sensitivity to heat and drought stress compared to storage proteins.^11^ The amylose synthesis is regulated by the 59‐kDa granule‐bound starch synthase (GBSS), (ADP‐glucose‐starch glycosyltransferase, EC 2.4.1.21), encoded by three waxy loci Wx‐A1, Wx‐B1 and Wx‐D1 located on chromosomes 4A, 7A and 7D, respectively. The combinations of three waxy proteins in eight wheat isolines indicate that amylose content and starch pasting properties are primarily determined by the absence of Wx‐B1 protein, followed by the ‐D1 and ‐A1 proteins.^12^

The amylose content ranges from below 1% in waxy wheat to 29% in wild‐type wheat flour, with an ideal content of 21–24% for Asian noodles.^13^ The waxy wheat starches have a higher water retention capacity than their regular counterparts. Increasing the ratio of waxy wheat starch in the reconstituted flours decreases the hardness of cooked noodles, even with a higher protein content.^14^ YAN, made from near‐isogenic wheat lines with a lower level of amylose content because of a deficiency in waxy (Wx) protein, is found to have relatively lower firmness but higher elasticity and smoothness compared to wild‐type wheat.^3^

Research indicates that Australian wheat cultivars suitable for making white‐salted udon noodles have a high flour swelling volume (FSV), which is linked to the GBSS‐4A null mutation. This mutation leads to a reduced amylose content and causes variations in the starch structure.^15^ Australian Prime Hard (APH) wheat, which has a protein content above 13% (11% moisture basis), is regarded as superior for producing ramen with an elastic texture because of its starch properties.^3^ However, the use of APH, primarily produced in Queensland and New South Wales, has decreased over time in Japanese ramen production as a result of limited supply. It would be useful if Australia could provide an alternative wheat option for producing high‐quality ramen noodles in Japan.

In the present study, we aimed to investigate the suitability of 32 Australian hard wheat cultivars grown on the Australian east coasts across three seasons with AH2 protein content ranging from 11.6 to 12.4%. The null4A or wild‐type (WT) hard wheat population has either an absence or presence of GBSS‐4A, and they are assessed in Japanese ramen noodles. We hypothesise that there might be variability in physicochemical properties among the WT and null4A hard wheat varieties with AH2 protein content, which in turns offer opportunities to select wheat varieties which might be better preferred for ramen sensory attributes.

## MATERIALS AND METHODS

### Samples

Table 1 shows that the Australian hard varieties population comprises protein content between 11.8 and 12.6%, test‐milled at 60% extraction rates using an MCKA Buhler test mill (Buhler, Uzwil, Switzerland). The 32 Australian Hard samples were harvested from 2021, 2022 and 2023 from either Queensland (QLD) or Northern New South Wales (NNSW) or South Australia (SA), with eight of them identified as WT and 24 as null4A (Table 1). Unlike the 2022 and 2023 samples, the 2021 samples were made up of only those harvested from QLD/NNSW.

**Table 1:** List of sample genetics made up of either wild‐type (WT) or null4A, along with the variety name, growing region of either from Queensland/Northern New South Wales or South Australia (SA), and harvest year

No.	WT/null4A	Variety	Growing region	Harvest year
1	WT	Janz	QLD/NNSW	2021
2	WT	Sunvale	QLD/NNSW	2021
3	null4A	Catapult	QLD/NNSW	2021
4	null4A	Coota	QLD/NNSW	2021
5	null4A	EGA Gregory	QLD/NNSW	2021
6	null4A	Hammer	QLD/NNSW	2021
7	WT	LRPB Lancer	QLD/NNSW	2022
8	null4A	Coota	QLD/NNSW	2022
9	null4A	Sunmax	QLD/NNSW	2022
10	null4A	Calibre	SA	2022
11	null4A	Cutlass	SA	2022
12	null4A	Hammer	SA	2022
13	null4A	Mace	SA	2022
14	null4A	Scepter	SA	2022
15	WT	Leverage	QLD/NNSW	2023
16	WT	Mitch	QLD/NNSW	2023
17	WT	Sundancer	QLD/NNSW	2023
18	WT	Beckom	QLD/NNSW	2023
19	null4A	Calibre	QLD/NNSW	2023
20	null4A	Coolah	QLD/NNSW	2023
21	null4A	Cutlass	QLD/NNSW	2023
22	null4A	Scepter	QLD/NNSW	2023
23	null4A	Var A	QLD/NNSW	2023
24	null4A	Sunblade	QLD/NNSW	2023
25	null4A	Suncentral	QLD/NNSW	2023
26	null4A	Sunmaster	QLD/NNSW	2023
27	null4A	Suntop	QLD/NNSW	2023
28	null4A	Vixen	QLD/NNSW	2023
29	null4A	Ballista	SA	2022
30	WT	Beckom	SA	2023
31	null4A	Mace	SA	2023
32	null4A	Scepter	SA	2023

### Physicochemical property assessments

The wheat protein and moisture contents were measured using a FOSS Infratec NIR machine (Foss A/S, Hillerød, Denmark). The falling number method was referred to AACC56‐81.03 with slight modifications. The flour protein, moisture and ash content were measured according to AOAC 992.23 and the Leco Manual (LECO Corp., St Joseph, MI, USA), AOACI 925.10, and AACC 08‐01.01, respectively. The starch damage and gluten content were referred to AACC 76‐30.02 and AACC 38‐12.02 and ICC 158, ISO 21415. The farinograph, extensograph and amylograph were carried out using RACI Official Testing Methods.^8^ FSV analyses and rapid viscosity analyses (RVA) were performed according to AACC56‐21.01 and 76–21.1, respectively. The amylose and total starch content were assessed by AACC 61–03.01 and AACC 76–13.01, and the amylopectin content was amylose content subtracted from the total starch content.

### Ramen noodle making and sensory evaluation

The ramen noodles were made by combining 500 g of flour with addition of 32% water and 1% of Kansui (sodium carbonate: potassium carbonate = 40:60) and 1% table salt in a Hobart mixer. The noodle dough was passed through noodle machine to achieve 1.4 mm thickness × 1.5 mm width × 20 cm length. Noodle was cooked under atmospheric pressure for 2.5 min and served with hot soup immediately.

The ramen sensory texture assessment was conducted by seven NIPPN trained panellists. The hardness (10), elasticity (20), surface smoothness (10), taste (10) and texture deterioration after cooking (20) of ramen were assessed, respectively. The sensory aspects, such as elasticity and texture deterioration, had doubled the weights, with more importance than firmness and surface smoothness. The taste of noodle was disregarded because all samples were considered to have the same level of desirable taste. Therefore, the total score of ramen sensory texture assessment was 60.

The APH was used as the reference control. The sensory scores for the APH control were hardness (7), elasticity (14), surface smoothness (7) and texture deterioration after cooking (14). Table 2 presents the physicochemical properties of the APH control.

**Table 2:** Physicochemical properties of APH reference control used in sensory evaluation

Test weight [10 kg m^3(−1)^]	81.4
Thousand kernel wt (g kg^−1^)	348.0
Wheat protein (g kg^−1^)	132.0
Wheat moisture (g kg^−1^)	106.0
Flour Protein (g kg^−1^)	110.0
Ash (g kg^−1^)	4.0
Moisture (g kg^−1^)	135.0
Water absorption (g kg^−1^)	604.0
Development time (min)	6.1
Extension area (cm^2^)	144.0
Extension length (cm)	14.4
Maximum resistance (BU)	693.0
Amylograph peak (BU)	622.0

### Statistical analysis

Statistical analyses were performed using Prism, version 10 (GraphPad Software Inc., San Diego, CA, USA). Significant differences between mean values were calculated based on two independent evaluations (*n* = 2), unless stated otherwise. Welch's *t*‐test and ordinary one‐way analysis of variance (ANOVA) were used to assess differences between the samples. *P* < 0.05 was considered statistically significant. Pearson correlation was conducted at a 95% confidence interval.

## RESULTS

### Physicochemical properties

Table 3 tabulates the physicochemical properties of all wheat samples. There were significant differences between samples tested for all traits except for flour ash content, wet gluten, set back and peak time.

**Table 3:** Physicochemical properties of wheat samples (*n* = 32)

Testing	Minimum	Maximum	Range	Mean	SD	SE	One‐way ANOVA F	*P* value	*P* value summary
Test Weight [10 kg m^3(−1)^]	78.4	84.7	6.3	81.7	1.7	0.3	93.7	<0.0001	****
Thousand Kernel weight (g kg^−1^)	335.0	512.0	177.0	394.9	45.9	8.1	18.7	<0.0001	****
Wheat protein (g kg^−1^)	115.8	129.2	13.4	123.5	4.2	0.7	34.7	<0.0001	****
Wheat moisture (g kg^−1^)	103.1	131.4	28.3	115.2	6.6	1.2	138.6	<0.0001	****
Particle size index	7.0	15.0	8.0	11.3	2.1	0.4	3.9	0.0001	***
Falling number (s)	406.0	530.0	124.0	455.0	33.1	5.8	4.3	<0.0001	****
Flour protein (g kg^−1^)	100.0	118.0	18.0	110.2	5.1	0.9	4.0	<0.0001	****
Moisture (g kg^−1^)	127.0	146.0	19.0	138.3	5.1	0.9	3.2	0.0007	***
Ash (g kg^−1^)	3.6	5.5	1.9	4.5	0.4	0.1	1.6	0.1024	NS
Starch damage (%)	6.1	8.7	2.6	7.5	0.8	0.1	33.2	<0.0001	****
Wet gluten (g kg^−1^)	231.0	318.0	87.0	286.5	2.6	0.5	0.5	0.956	NS
Gluten index	77.0	99.6	22.6	94.8	5.2	0.9	2.2	0.0171	*
Colour Minolta L*	92.1	94.4	2.3	92.9	0.7	0.1	37.8	<0.0001	****
Colour Minolta a*	−0.6	0.3	0.9	−0.2	0.2	0.0	4.6	<0.0001	****
Colour Minolta b*	7.4	12.7	5.3	9.3	1.4	0.2	177.3	<0.0001	****
Peak viscosity (RVU)	152.0	258.0	106.0	213.2	27.7	4.9	24.0	<0.0001	****
Hold viscosity (RVU)	103.0	155.0	52.0	130.7	11.4	2.0	28.6	<0.0001	****
Breakdown (RVU)	31.0	121.0	90.0	82.4	21.7	3.8	37.7	<0.0001	****
Final viscosity (RVU)	188.0	262.0	74.0	234.4	16.7	3.0	3.6	0.0003	***
Set back (RVU)	85.0	121.0	36.0	103.6	9.4	1.7	0.6	0.9138	NS
Peak time (min)	5.7	6.1	0.4	5.9	0.1	0.0	0.2	>0.9999	NS
Water absorption (g kg^−1^)	551.0	674.0	123.0	598.5	25.6	4.5	3.3	0.0006	***
Development time (min)	2.3	11.3	9.0	6.8	1.8	0.3	17.7	<0.0001	****
Stability time (min)	7.2	25.2	18.0	15.7	5.4	1.0	5.7	<0.0001	****
Extension area (cm^2^)	74.0	213.0	139.0	156.7	33.3	6.0	18.4	<0.0001	****
Extension length (cm)	18.0	26.3	8.3	22.4	2.0	0.4	3.4	0.0005	***
Maximum resistance (BU)	262.0	761.0	499.0	529.2	106.5	19.1	56.7	<0.0001	****
Amylograph peak (BU)	385.0	925.0	540.0	642.5	135.5	25.6	2.2	0.0229	*
Amylopectin (g kg^−1^)	432.0	541.0	109.0	488.3	30.7	5.4	8.4	<0.0001	****
Amylose (g kg^−1^)	156.0	230.0	74.0	191.9	18.3	3.2	6.7	<0.0001	****
Total starch (g kg^−1^)	631.0	719.0	88.0	682.4	25.4	4.5	5.7	<0.0001	****
Flour swelling volume (mL g^−1^)	15.8	21.6	5.8	19.4	1.8	0.3	139.4	<0.0001	****

NS, not significant.

### Comparing physicochemical properties of WT and null4A hard wheat varieties

Table 4 compares the physicochemical properties of hard wheat varieties made up of WT against those of null4A genetic basis. Only test weight, thousand kernel weight, peak viscosity, amylose content and FSV were significantly different from each other.

**Table 4:** Comparison of physicochemical properties of hard wheat varieties made up of wild type and null4A genetic basis

Testing	Wild type (*n* = 8)		null4A (*n* = 24)		*t*‐test	
	Mean	SD	Mean	SD	*P* value	*P* value summary
Test weight (10 kg m^3(−1)^)	82.3	2.0	81.6	1.6	0.0000	****
Thousand Kernel weight (g kg^−1^)	365.4	26.3	404.8	47.1	0.0000	****
Wheat protein (g kg^−1^)	124.4	4.5	123.2	4.1	0.6185	NS
Wheat moisture (g kg^−1^)	114.3	4.8	115.5	7.1	0.3912	NS
Particle size index	11.5	2.1	11.2	2.1	0.7324	NS
Falling number (s)	451.0	44.6	456.3	29.4	0.7605	NS
Flour protein (g kg^−1^)	112.5	0.5	10.9	0.5	0.0736	NS
Moisture (g kg^−1^)	136.6	6.6	138.9	4.5	0.4634	NS
Ash (g kg^−1^)	4.38	0.41	4.59	0.45	0.2695	NS
Starch damage (%)	7.0	0.8	7.7	0.8	0.0532	NS
Wet gluten (g kg^−1^)	290.0	24.0	285.4	27.0	0.6292	NS
Gluten index	96.3	2.4	94.3	5.8	0.1805	NS
Colour Minolta L*	93.2	0.7	92.8	0.6	0.1762	NS
Colour Minolta a*	−0.2	0.2	−0.2	0.2	>0.999999	NS
Colour Minolta b*	8.7	0.5	9.5	1.6	0.0394	NS
Peak viscosity (RVU)	185.0	20.2	222.6	23.3	0.0007	***
Hold viscosity (RVU)	126.3	8.0	132.2	12.0	0.1320	NS
Breakdown (RVU)	58.8	18.5	90.3	16.5	0.0013	NS
Final viscosity (RVU)	239.1	14.0	232.9	17.6	0.3267	NS
Set back (RVU)	112.9	8.7	100.5	7.5	0.0043	NS
Peak time (min)	5.9	0.1	6.0	0.1	0.0306	NS
Water absorption (g kg^−1^)	594.8	24.1	599.8	26.5	0.6258	NS
Development time (min)	6.9	1.4	6.7	1.9	0.7545	NS
Stability time (min)	17.0	4.6	15.3	5.7	0.4088	NS
Extension area (cm^2^)	160.6	26.7	155.3	35.8	0.6630	NS
Extension length (cm)	23.3	1.2	22.0	2.1	0.0425	NS
Max resistance (BU)	517.4	69.2	533.3	117.8	0.6478	NS
Amylograph peak (BU)	501.0	113.1	673.3	121.1	0.0029	NS
Amylopectin (g kg^−1^)	469.6	25.1	504.3	53.7	0.0234	NS
Amylose (g kg^−1^)	209.7	14.1	185.1	16.3	0.0009	***
Total starch (g kg^−1^)	683.3	20.6	682.1	27.2	0.9153	NS
Flour swelling volume (mL g^−1^)	17.0	0.8	20.2	1.3	<0.000001	****

NS, not significant.

### Effect of amylose content in physicochemical properties

**Figure 1:**
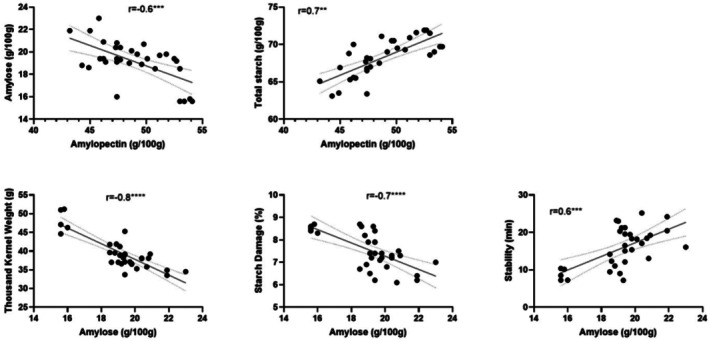
Pearson correlation of amylose and amylopectin with physicochemical properties of AH population (**P < 0.01; ***P < 0.001; ****P < 0.0001).

Figure 1 demonstrates that the amylopectin content was negatively correlated with amylose content but positively with total starch. The amylose content was negatively correlated with thousand kernel weight, starch damage and positively correlated with dough stability.

### Correlation of wheat quality parameters

**Figure 2:**
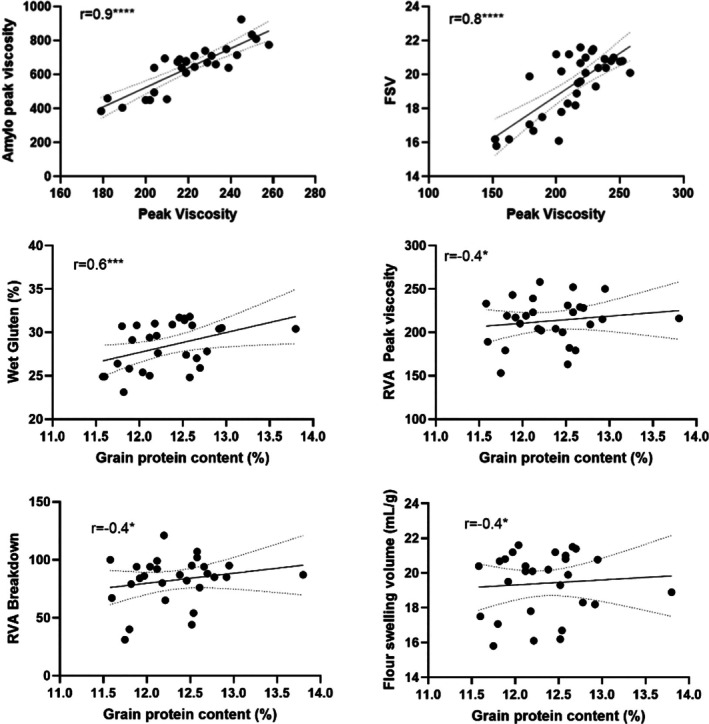
Pearson correlation of peak viscosity with amylograph peak viscosity and flour swelling volume (FSV) and grain protein content *versus* wet gluten, rapid viscosity analyses (RVA) breakdown, peak viscosity and FSV (****P < 0.00001; ***P < 0.001; *P < 0.05).

Figure 2 shows that peak viscosity was highly correlated with both Amylography peak viscosity and FSV, and the grain protein content correlates mildly, although significantly with wet gluten. The grain protein content was significantly correlated with peak viscosity, breakdown and FSV, but their correlations were quite variable.

**Figure 3:**
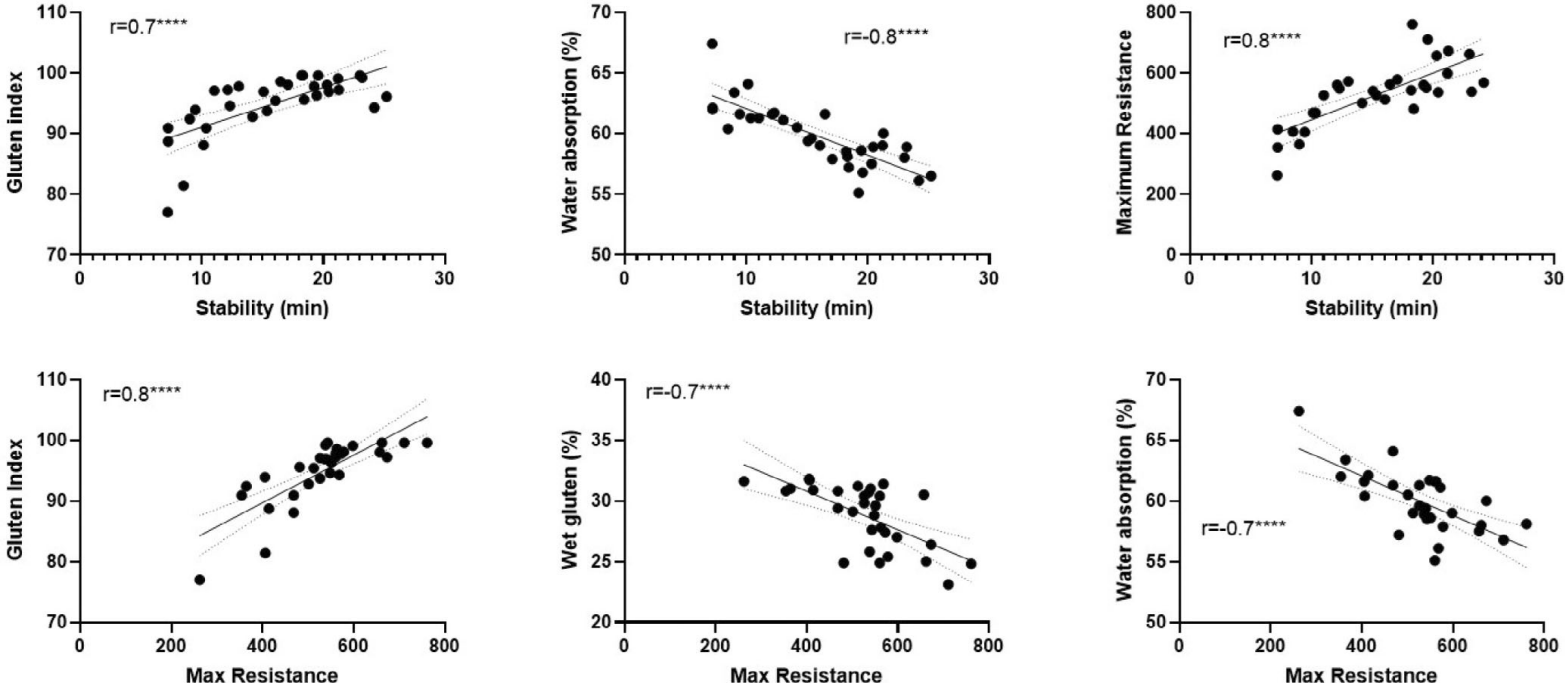
Pearson correlation of stability and max resistance with gluten index, wet gluten and water absorption (****P < 0.0001).

The dough stability was positively correlated with gluten index but negatively correlated with water absorption and maximum resistance (Fig. 3). The dough maximum resistance was positively correlated with gluten index, but negatively correlated with wet gluten and water absorption.

### Ramen sensory texture and colour assessment

**Figure 4:**
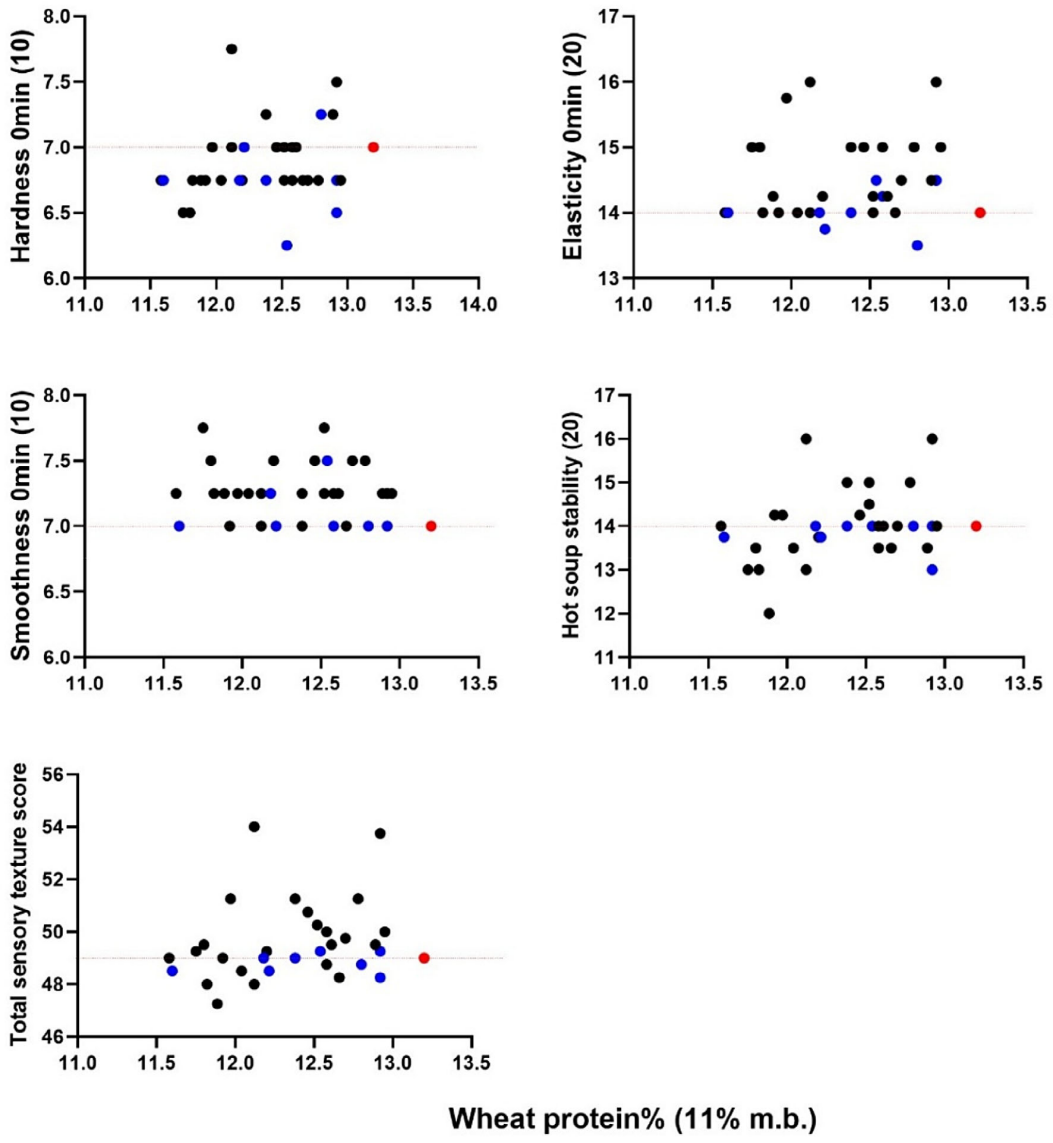
Comparison of sensory noodle hardness, elasticity, surface smoothness, hot soup stability, total sensory texture score, noodle sheet colour and specks of AH varieties null4A (black) and wild‐type (WT) (blue) against APH (red).

Figure 4 shows the noodle sensory elasticity and total sensory texture score between null4A, and WT was significantly different. Oppositely, the sensory hardness, surface smoothness, hot soup stability, noodle sheet colour and specks were not significantly different.

**Figure 5:**
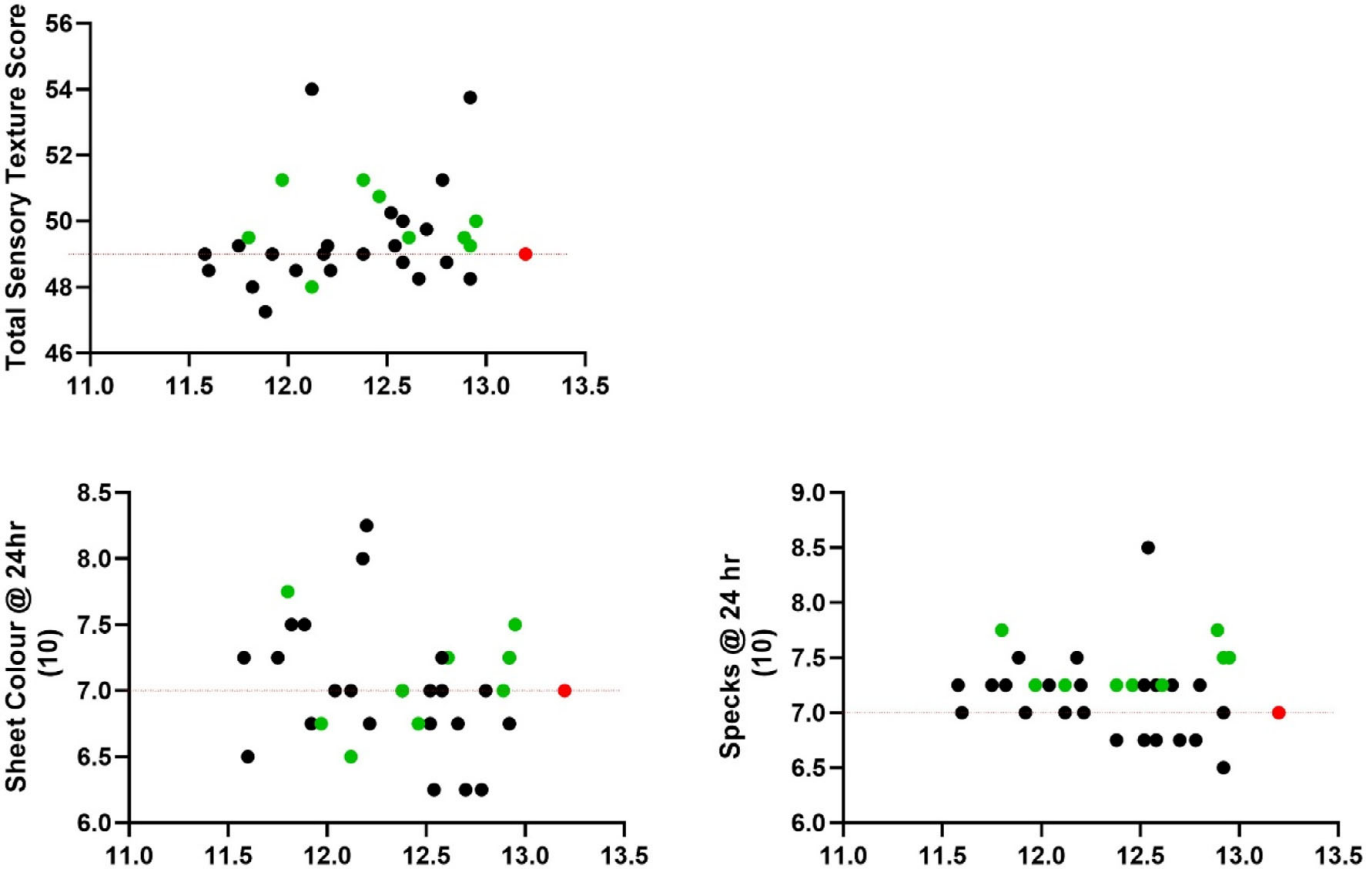
Distribution of total sensory texture score, raw noodle sheet colour and specks of AH varieties grown in Queensland/New South Wales (QLD/NNSW) (black) and South Australia (SA) (green) compared to APH (red).

Figure 5 shows that most AH population from SA was rated as a higher total sensory texture score compared to APH. About half of the AH population from QLD/NNSW, and all of those from SA, outperformed APH in sensory raw noodle colour and specks assessments, respectively.

### Correlation of noodle sensory texture and physicochemical properties

**Figure 6:**
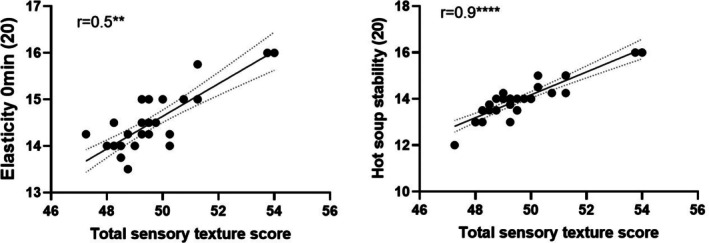
Pearson correlation of noodle sensory elasticity and hot soup stability with total sensory texture score.

The sensory noodle elasticity is shown to correlate mildly, whereas the noodle hot soup stability correlates strongly with total sensory texture score (Fig. 6). There were no other notable correlations between sensory noodle total texture score and breakdowns with other physicochemical properties.

## DISCUSSION

### Hard wheat varieties with a narrow protein range of 11.6–12.9% had differing physicochemical properties

The wheat protein content of hard wheat varieties ranged from 11.6% to 12.9%, which is within the range of Australian Hard (AH) 2 grade.^16^ Within the narrow grain protein range, the physicochemical properties of the 32 hard wheat varieties are significantly different from each other, reflecting the notable variability among the AH population, except for flour ash content, set back and peak time. The average flour protein and ash contents of AH varieties are 11.0% and 0.36%, respectively, which matches with the commercial Japanese alkaline noodle flour as reported by Jun *et al*..^17^ However, the starch damage of AH population was at 7.5% and it was higher than that of 5.6%, which is likely affected by test‐milling.

Japanese ramen is traditionally made from wheat with a relatively higher protein content, with up to 3% higher than udon noodles.^18^ However, a higher protein content is known to contribute to a darker noodle colour, which is not favoured and made worse by wheat with red instead of white seed coat.^2^ Similar to all Australian milling wheat, the AH population is white seed which is preferred over the red seed counterpart for Japanese ramen production from the aspect of colour appearance.^18^

### Comparison of null4A and WT AH population

Japanese wheat varieties that have a lower level of starch‐granule bound protein are associated with a reduced amylose level.^19^ This reduction is influenced by the partial deletion of proteins, specifically null alleles for the waxy protein in wheat grain, which supports that the null4A AH population exhibited a lower amylose content compared to that of WT.

The wheat protein, moisture, particle size index and falling number are not different between the null4A and WT populations. However, the test weight of the null4A population was slightly lower, and its thousand kernel weight was higher than that of WT, indicating that physical grain properties could be affected by the null4A genetic basis. The grain protein and thousand kernel weight could be affected by genotype and growing environment.^20^ Although thousand kernel weight between the two populations differ, their test weight is considered quite similar with a comparable grain hardness. Particle size index is directly correlated with grain hardness and it is known to influence the behavior of fracture and the mechanical properties of whole wheat grain and endosperm during the milling process,^21^ where both populations are expected to have similar milling quality.

Interestingly, the flour protein content and other physicochemical properties were not significantly different from each other, except for peak viscosity, amylose content and flour swelling volume. Our findings indicate that null4a mainly contributes to a higher pasting viscosity with its starch granules having a relatively higher swelling property, creating a more viscous slurry with greater water‐holding capacity when cooking, which could be affected by a lower amylose content and a relatively higher amylopectin content. This is in line with the report by Yamamori and Quynh^12^ showing that GBSS affects production of amylose content and pasting properties in Japanese wheat varieties. However, the peak viscosities of the Australian wheat cultivars made up of 10 null4A and 25 normal for the GBSS gene on chromosome 4A are not significantly different from each other, but the hold and final viscosities and the breakdown of the two populations are significantly different as reported by Ross *et al*.,^4^ which disagreed with our findings. This observation may suggest that their null4A population is influenced by a higher level of enzymatic activity, along with a somewhat lower protein content.

### Relationships of amylose and amylopectin contents with flour functionalities

Our results suggest that the reduction in amylose content in the AH population may contribute to a higher amylopectin content, thousand kernel weight and starch damage, but it does not affect the total starch content. Conversely, a higher amylopectin content directly contributes to a higher total starch content. This observation is generally supported by what reported by Chen *et al*.^22^ and Rakszegi *et al*.^23^ where the reduced level in amylose content slightly affects the synthesis of amylopectin but greatly reduced the biosynthesis of total starch and grain weight. The positive relationship between amylopectin and total starch content in the present study might be better highlighted by the AH population with a relatively narrow protein range.

Although the amylose content of AH was significantly correlated with starch damage and stability, the results are skewed by the separation of the two distinct populations, where the smaller population is made up of five varieties harvested from SA in 2022–2023, suggesting amylose content could be affected by growing location, as confirmed by our results. Our findings show that the amylose and amylopectin contents affect grain weight and total starch content respectively, but not other flour functionalities.

There is significant variation among the AH population in flour functionalities, and they are not directly correlated with either amylose or amylopectin contents. The overall results suggest that null4A genetic basis contributes to a relatively lower amylose content and a higher peak viscosity and FSV. However, they do not have a direct correlation, suggesting that the variation in flour functionalities among the AH population could be governed by other genetic factors beyond the WxB1 genes. The variation could be contributed by concerted genetic factors as a result of different combinations or types of null alleles of GBSS affecting the synthesis of amylose content,^12^, ^24^ the variation in amylopectin synthesis through a combined reactions catalysed by ADP‐glucose pyrophosphorylase, starch synthase, starch‐branching enzyme and starch‐debranching enzyme,^22^ and other wheat gluten proteins.^25^

### Flour pasting and gluten qualities

The peak viscosity measured by RVA is highly and positively correlated with peak viscosity measured by an amylograph and FSV analyses. FSV analysis was developed by Crosbie *et al*.^26^ to identify wheats potentially suitable for Japanese udon type noodles. It measures the swelling volume of flour reflecting the extent of the water uptake by starch during gelatinisation and it is known to be strongly correlated with starch paste peak viscosity,^27^ as agreed by our findings. The samples achieving a high FSV correlate with a high total texture score made up of softness/hardness, elasticity and smoothness. Our results suggest that the peak viscosity, whether measured by RVA or an amylograph, and FSV analysis provide similar information on the starch characteristics of the flour samples.

The grain protein content of AH population was significantly correlated with wet gluten, peak viscosity, breakdown and FSV. However, wet gluten is not significantly different among the AH varieties and the correlations between protein content and peak viscosity, breakdown and FSV are unreliable and should not be taken into account. This further emphasises that the differences in physicochemical properties between the AH population are unlikely to be influenced by protein content. In a similar vein, although there are correlations between starch damage and other aspects such as thousand kernel weight, particle size index, gluten index, final viscosity, setback, water absorption, stability, area and extensograph length, these correlations are considered weak therefore not regarded (results not shown).

Wheat categorised as elite or having a stronger gluten strength tends to have a higher gluten index, wet gluten, water absorption, maximum resistance, development and stability, usually driven by a higher protein content containing a higher level of gluten content, gliadins, glutenins and glutenin macropolymer.^28^, ^29^, ^30^ It is interesting that, in the present study, although the AH population has a relatively higher gluten strength with a higher maximum resistance, stability and gluten index, these contribute to a lower wet gluten content and water absorption. The inverse relationship between gluten strength and gluten content and water absorption might be highlighted by the narrow protein range among the AH population.

### Effect of growing location and year

The varieties of wheat grown in QLD/NNSW and SA were largely dissimilar. Additionally, different varieties were cultivated in various locations and across different years. Therefore, comparing the effects of growing locations and years on the physicochemical properties of wheat is not meaningful. However, it is noteworthy that, unlike those in QLD/NNSW, the wheat varieties grown in SA primarily have a null4A genetic background.

It is widely recognised that ash content typically has a negative correlation with noodle colour and the appearance of specks. In this context, we found it noteworthy that all SA samples, primarily derived from the null4A genetic background, were preferred over the APH and WT counterparts because of their lower level of speck appearance, despite having a relatively higher ash content (results not shown). We believe this observation may be influenced by various factors, including genetic makeup or specific growing conditions related to the year. Further investigations would be beneficial to gain a deeper understanding of these findings

### Relation of noodle sensory texture to other physicochemical properties

The grain protein content and other physicochemical properties of AH population are not directly correlated with the total and breakdown of sensory texture scores of the AH population. Although protein content, dough strength and starch paste viscosity are known to affect noodle texture, there is an optimal range to achieve desirable noodle sensory quality, with quality decline occurring outside of this range.^8^ Generally, the protein content contributes to increased sensory firmness, but it reduces surface smoothness. This explains the reason the AH population has a higher surface smoothness regardless of null4A or WT genetic background as a result of a lower protein content than APH.

Among the physicochemical properties, only the peak viscosity, amylose content and FSV were significantly different among the null4A and WT groups. The elasticity and total noodle sensory texture score for null4A were higher than WT. This signifies that preferred sensory texture delivered by null4A population is likely contributed from a higher peak viscosity, lower amylose and higher FSV. However, these relationships were not linear as noted, which are likely attributed to variation in null4A population governed by other genetic factors aside from their protein content and pasting properties.

The noodle sensory elasticity and hot soup stability carrying double the weight and importance than hardness and surface smoothness, thereby having a more direct impact on total noodle sensory texture score. The sensory hardness and hot soup stability were not significantly different between null4A and WT. Even though the median between the two groups were the same, the null4A achieves a much wider range with a slightly higher mean values, indicating more variation exists. Conversely, the number of WT varieties was smaller, and their total sensory texture score was generally like or slightly lower than that of APH, suggesting that APH is likely made up of varieties with a nature closer to WT varieties.

Interestingly, up to 61% and 39% of the AH population exhibited hot soup stability and firmness that were like or higher than those of the APH control, respectively. Despite having significantly lower protein content, some of the AH varieties exhibit a comparable or higher sensory noodle firmness and hot soup stability comparable to or even greater than that of the APH population. Strong‐gluten wheat flour and the addition of 15% waxy wheat flour is shown to improve noodle sensory firmness and springiness as a result of waxy wheat starch causing a greater formation of strong crystalline structure of amylopectin, whereas waxy wheat gluten results in a greater level double‐helical short‐range molecular structure of starch.^31^ The hot paste viscosity is reportedly more stable for waxy wheat containing Wx‐D1f, which could be the result of a higher level of extra‐long chains of amylopectin and or the low‐molecular‐weight amylose.^32^ Therefore, the interaction between starch pasting property associated with the variation among the AH population with null4A genetic background, in combination with the variation of gluten qualities, could be contributing to ramen with preferred overall sensory texture with high firmness, elasticity, surface smoothness and hot soup stability.

## CONCLUSIONS

Our overall results show Australian Hard (AH) wheat with a narrow protein range of 11.6 to 12.9% with null4A genetic background offers a varied range of physicochemical contributing to variation in Japanese ramen sensory properties. The null4A population has relatively higher pasting properties, likely attributed to a lower level of amylose content, resulting in ramen with overall more preferred sensory attributes, particularly in elasticity, compared to its wide‐type counterpart. The AH population cultivated in SA has a relatively higher ash content compared to those of cultivated in QLD and NSW; however, it does not necessarily contribute to a less preferred raw noodle sheet colour and specks appearance. A greater proportion of null4A population has been shown to offer variation in noodle sensory qualities, suggesting that there is potential to select certain hard wheat varieties within the narrow protein range of AH2 for high quality ramen offering a combination of high elasticity, surface smoothness, firmness and hot soup stability that could be appreciated by the Japanese market. Selected hard wheat varieties with an AH2 protein range, primarily derived from a null4A genetic background, may provide an alternative to APH for producing ramen noodles favoured by the Japanese market.

## Data Availability

The data that support the findings of this study are available from the corresponding author upon reasonable request.
